# Navigating Barriers to Oral Health Challenges Faced by Children With Autism Spectrum Disorder: A Scoping Review

**DOI:** 10.7759/cureus.66493

**Published:** 2024-08-09

**Authors:** Joice Alexander, Sibyl Siluvai, Ajay Mathew George, Indumathi K. P., Victor R Lazar, Nandita Kshetrimayum

**Affiliations:** 1 Public Health Dentistry, SRM Kattankulathur Dental College and Hospital, SRM Institute of Science and Technology (SRMIST), Chengalpattu, IND; 2 Radiodiagnosis, SRM Medical College Hospital and Research Center, SRM Institute of Science and Technology (SRMIST), Chengalpattu, IND; 3 Public Health Dentistry, Regional Institute of Medical Sciences, Imphal, Imphal, IND

**Keywords:** disability, dental challenges, barriers, oral health care, children, autism spectrum disorder

## Abstract

This article identifies the multifaceted challenges that hinder optimal oral health among children diagnosed with autism spectrum disorder (ASD). While dental care is a fundamental aspect of overall well-being, children with ASD encounter unique obstacles that often go unnoticed. Drawing from a comprehensive review of existing literature and insights from healthcare professionals, this article explores the sensory sensitivities, communication difficulties, and behavioral issues that contribute to suboptimal oral hygiene in this demographic. We also discuss the critical role of caregivers, dentists, and educators in addressing these challenges, emphasizing the importance of early intervention and tailored strategies to improve the oral health of children with ASD. By shedding light on these obstacles, this article aims to foster a more inclusive and holistic approach to oral healthcare for children with ASD, ultimately enhancing their overall quality of life.

## Introduction and background

The word autism derives from the Greek words "autos," which means "self," and "ismos," which denotes a state of being [1]. The term autism spectrum disorder (ASD) describes a collection of early-appearing social interaction challenges and repeated motor and sensory behaviors that are additionally accompanied by a significant hereditary component, in addition to other characteristics [2]. Over the years, society's perception of autism has changed. Autism is increasingly understood to be a neurodevelopmental difference rather than a disease or flaw.

More frequently than previously thought, mental retardation is now recognized as one of the most prevalent developmental disorders, followed by ASD [3]. In contrast to prevalence estimates for autism in the 1960s, when the first systematic research was carried out, current prevalence estimates for the complete autism spectrum are approximately 60 per 10,000. This 15-fold increase has generated concerns about an outbreak [4]. The Centers for Disease Control and Prevention (CDC) in the United States estimate that 1 in 36 American children suffers from ASD [5]. King Abdulaziz City for Science and Technology figures indicate that 1 in 180 Saudi children has an ASD diagnosis [6]. Increased diagnostic facilities, awareness, and specialized services may be to blame for the significantly increased prevalence of diagnosed cases.

A topic that has been determined to be of high priority for intervention is known as a leading health indicator. Public health is seriously affected by oral health issues [7]. People in underdeveloped countries have unacceptably high rates of dental problems, notably decayed teeth and periodontal issues, despite recent improvements in oral health among autism patients and the general population worldwide [8]. In many nations, the uneven use of dental care is influenced by socioeconomic position [9]. The Federation Dentaire Internationale categorizes barriers to accessing dental services into two groups: (a) people themselves (including a shortage of perceived need, anxiety and fear, cost constraints, and poor access); and (b) dentists (improper facilities, inconsistency in resources, and training unsuitable for changing needs and demands). Among the social problems society faces are a lack of public support for behaviors that promote health, a lack of oral healthcare facilities, a lack of workforce planning, and a lack of research support [10-12]. 

Autism is a neurological disorder that affects behavior, speech, and social interaction. People with ASD may have sensory sensitivity, struggle with changes, and struggle to understand and communicate their needs. These elements may make their oral hygiene regimens and dentist visits extremely difficult.

Children with ASD face many obstacles, but most of them go unaddressed. The first step in solving any issue is gaining a thorough understanding of it, which, when accomplished by a methodical analysis of the available literature, constitutes the highest level of scientific proof. A comprehensive evaluation was carried out to assess the oral health difficulties that children with ASD encounter.

## Review

Methods

Study Design

A detailed review was conducted to locate, assess, and compile all significant study findings according to PRISMA Extension for Scoping Reviews (PRISMA-ScR) criteria. The following standards were created after the PICO analysis of the articles discovered during the search: the population included children with ASD; the interest was the challenges faced in maintaining good oral health; the comparison was not relevant for the study; and the outcome was the obstacles to maintaining healthy oral hygiene. In the PICO analysis, "Interest" was used in place of "intervention" because the study topic for this review is qualitative, and "Comparator" was not relevant. Table 1 depicts the types of studies that were included and excluded from this review.

**Table 1:** Inclusion and exclusion criteria

Inclusion criteria	Only studies published in English were considered in the search
	The study concentrated on papers that detailed the challenges faced by children with autism spectrum disorder in terms of oral health
	Children with autism spectrum disorder were the subjects of the studies
	Studies that focussed on Autism Spectrum Disorder were considered
	Studies that were published within the last fifteen years were included
	Cohort and cross-sectional study designs were taken into consideration
Exclusion criteria	Studies that revealed impediments to the general public's use of dental care
	Case studies, narrative reviews, and professional judgment
	Publications that were wholly unrelated to the subject of the investigation were excluded
	If translations into English were needed for studies

The study was organized based on its titles and abstracts. Studies whose abstracts satisfied all criteria for inclusion were then chosen for full-text reading. When a study met the eligibility criteria but the data in the abstract were inadequate, the full texts of the publications were also collected. Additional literature searches were conducted following the bibliographies of the finalized papers.

Search Method

Through MEDLINE (PubMed) and Cochrane, pertinent studies from the period of January 2008 to December 2022 were included since the study intended to include recent study outcomes. Using MeSH words, a thorough search strategy was created for MEDLINE and updated for PubMed and Cochrane. The Boolean operator OR is used to distinguish the terms "barrier to oral health" and "children with autism spectrum disorder" in the first group. The second group had the terms "challenges" and "specially-abled," while the third group contained the terms "children," "differently-abled," and "autism," with the Boolean operator "OR" separating them. Between June and August 2023, two authors, Dr. Joice Alexander and Dr. Sibyl Siluvai, conducted data searches to find more pertinent references and manual searches of the reference list. When studies similar to the topic were available in various databases, information from the studies was taken and reviewed. Search phrases and MeSH terms used for each database are shown in Table 2, as well as the total number of searches.

**Table 2:** Search terms

Database	Search structure
PubMed	(((barriers[All Fields] AND ((“utilization” AND (“dental care” AND (“autism spectrum disorder [MeSH Terms] OR (“dental”[All Fields] AND “care”[All Fields]) OR “dental care”[All Fields] OR “dental services”[All Fields]))) AND “ASD”[All Fields]) OR “Specially abled[All Fields])) OR ((“children”[MeSH Terms] OR “young population”[All Fields]))
Cochrane	ID-Search #1 - MeSH descriptor: dental care services #2 -Barriers #3 - MeSH descriptor: autism spectrum disorder #4 - children population #5 - children or child #6 - #1 and #2 #7 - #3 and #4 #8 - #5 and #6 and #7

Study Selection

Independently comparing titles and abstracts to the inclusion and exclusion criteria, the first and second authors determined which papers should be included in the study. The full-text papers that they were unable to reject based solely on title and abstract were then reviewed independently by the same two authors. There was no disagreement over inclusion in the two authors' comparison of the studies. The third author then cross-verified the final articles chosen. An inter-rater agreement of 0.8 was discovered.

Data Extraction

A data extraction form was used to gather the data from the final seven publications. It includes the name of the first author, the publication year, the study population, the objectives, the design, the method used to collect relevant data (using an assessment instrument), the primary finding, and the author's conclusion.

Results

Search Results

**Figure 1:**
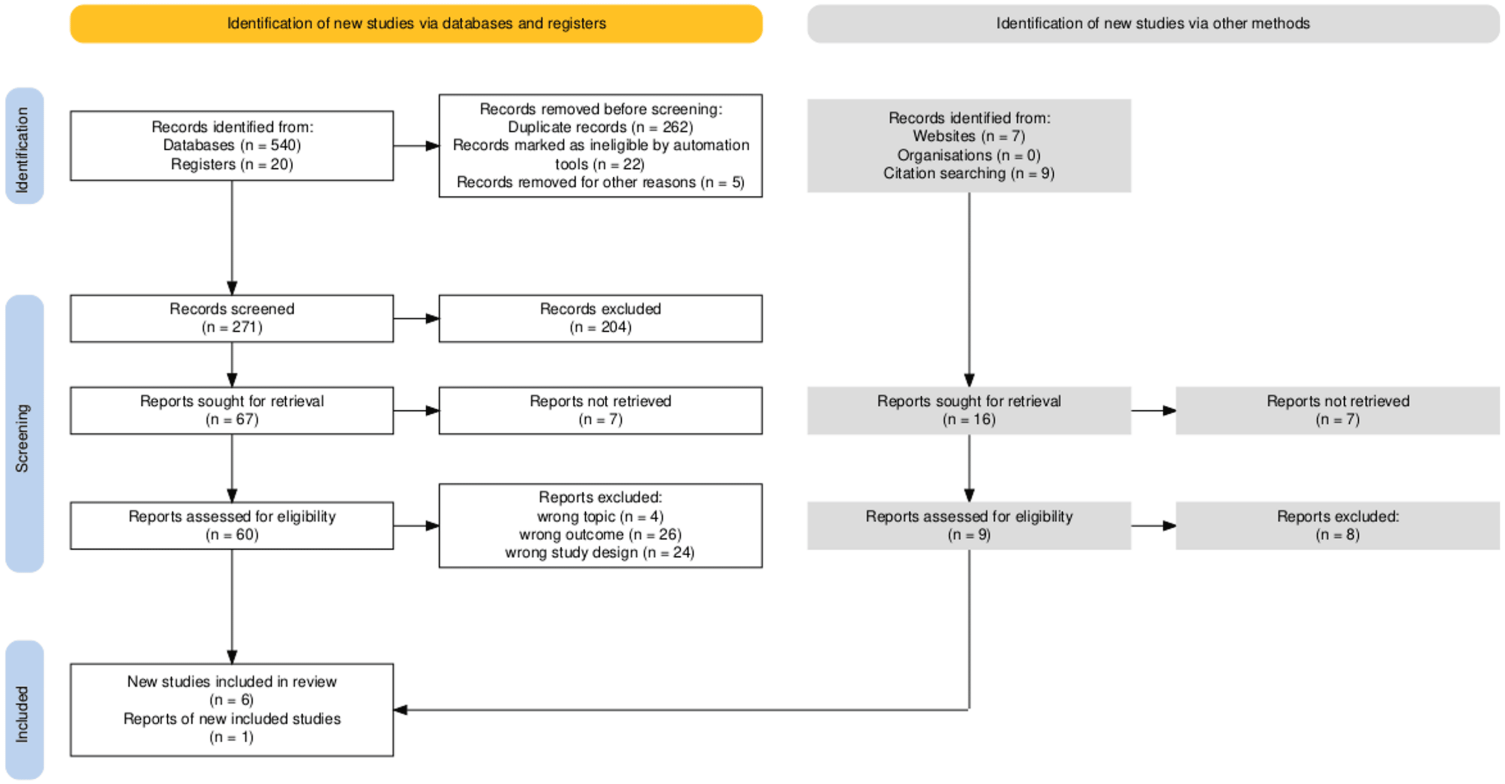
PRISMA-ScR standards for article selection PRISMA-ScR: Preferred reporting items for systematic reviews and meta-analyses extension for scoping reviews

A total of 576 pertinent papers were found in the search results from the electronic databases Cochrane and PubMed. Figure 1 depicts the PRISMA-ScR, standards for the article selection as a flowchart [13].

Main Results

A common theme among all the papers that were reviewed was the difficulty that children with ASD had in accessing dental care services in different parts of the world. Table 3 elaborates on the details of data extracted from the included studies.

**Table 3:** Extraction of data from included studies ASD: Autism spectrum disorder

Article reference	Location	Study aim	Study design and sample size	Key findings on challenges faced by children with ASD	Limitations
Alkhthami (2019) [14]	Old Dominion University	Influence of socioeconomic status, dental anxiety, cooperation of the child on the treatment of children with ASD	Questionnaire sample size: 200	Low household income	Sample size was not sufficient to draw conclusions
				Financial burden	Survey results may not be accurate as the data was collected from parents/guardians
				Lack of cooperation	No other medical conditions in the survey group were considered
				Difficulty finding a dentist with specialized training	Geographical limitation
				Lack of willingness by dental providers	
				Dental anxiety	
				Lack of experience	
Mansoor et al. (2018) [15]	United Arab Emirates	Challenges faced by ASD children and their families	Case-control sample size: 91	Avoid cleaning teeth	Lack of data on the total number of children with ASD in Dubai
				Disliking the way, the toothpaste and toothbrush feel	Data was collected via questionnaire and not a face-to-face interview, so lack of true information gathered from the parents
				Uncooperativeness	
				Finding a dentist who can treat a child with autism	
				The child cannot tolerate waiting in the waiting room area	
				Dental anxiety	
Du et al. (2019) [16]	Hong Kong	To identify and compare the oral health behaviors and barriers to oral health care among preschool children with and without ASD in Hong Kong. To evaluate the oral health knowledge and oral health attitudes among parents of preschool children with and without ASD	Questionnaire sample size: 257	Not being able to get a dentist who will provide treatment for the child	Small population
				Locating a dentist close to the kid's house	
				Anxiety or trepidation on the part of the dental personnel when treating the youngster	Geographical limitations
				Low family income	
				Low parent’s education	The accuracy of the response to the questionnaire cannot be evaluated
				Dental anxiety	
				Concerns about excessive fluoride ingestion	
				Gagging or intolerance	Most of the parents included in this study had high educational profiles and hence had more knowledge of the importance of oral care.
				Problems with manual dexterity and understanding brushing instructions	
				Lack of parental awareness	Geographical limitations
				High cost of dental treatment	
				Waiting for area ambiance	
				Long waiting duration	
				Audible disturbances coming from dentist offices or open cubicles	
				Hearing or seeing dental equipment or an operating room light	
Taghizadeh et al. (2019) [17]	Melbourne, Australia	To investigate the involvement of children with ASD undergoing a day procedure	One-on-one interview sample size: 29	Limitations in the staff's understanding of specific requirements	Limited study samples
				Protocol rigidity	Findings may be particular to the institution
				Lack of formal training	The responses may be biased
				Lack of explanation to patients and caregivers	
				Absence of adequate equipment	
				Beds that lower to ground level	
				Lack of suitable environment	
				Waiting time	
Alshihri et al. (2021) [18]	Riyadh, Saudi Arabia	To assess barriers faced influencing ASD children’s access to oral health care	Questionnaire sample size: 142	Cost	Any discrepancy in ASD diagnosis and reporting by parents
				Locating a dental clinic that will treat their ASD child	Limited study population
				The behavior of their ASD child	Geographical limitation
				Lack of cooperation	
				Mostly hypersensitive to touch and sounds	
Stein et al. (2012) [19]	California	To identify the challenges faced by children with ASD in accessing oral care in comparison to their typically developing peers	Questionnaire sample size of the study group: 196; a sample size of typically developing peers: 202	Hatred of the toothpaste's flavor or texture	Significant age difference in the study group
				Bright lights, loud noises, having objects put in one's mouth, lying back on the dentist's chair, odors, and dentist drilling were all things the young patient feared, despised, or complained about	
				Increase in uncooperative, sensory-sensitive, and self-stimulatory behaviors	The responses given by the caregivers and parents may be influenced or biased
				Being refused treatment by a dental provider	
Nithya et al. (2016) [20]	India	To identify the dental the challenges faced by children with ASD in accessing dental care	Questionnaire sample size: 31	Lack of awareness	Small study sample
				Behavioral issues	Geographical limitations
				Transport to the dentist	The reliability of the responses given for the questionnaire may be compromised
				Lengthy waiting hours	
				Dentist’s lack of knowledge and training	

Oral Health Status in Children With ASD

According to reports, children with developmental problems have the worst oral health and have a harder time accessing dental care than their neurotypical peers [21]. There is conflicting evidence from studies on whether oral disease is more prevalent in kids with ASD. Studies showed no differences in gingivitis, caries, or dental hygiene between kids with ASD and their peers [22,23]. Several studies have found that individuals with ASD are more susceptible to caries [24,25]. The study involved 61 patients who went to autism centers in Sharjah and Dubai. Children with ASD exhibited significantly worse periodontal health than normal children, and 97% of them had gingivitis [24]. In a different study that involved 32 individuals with ASD and was conducted in Bangkok, 78% of the participants experienced gum bleeding, necessitating professional cleaning in 71.9% of the cases [23]. In addition, a study in Southern Illinois of 55 children with ASD discovered that 62% of them had obvious gingivitis [26].

Patients with ASD frequently display oral behaviors that are detrimental to their dental health. Children with ASD may have unfavorable oral habits, like lip biting, tongue thrusting, and gingival poking. Crowding and an open bite are common malocclusions seen in children with ASD [27]. As a result of their extreme maxillary crowding, posterior crossbite, and increased overjet, children with ASD are especially prone to oral health issues such as dental caries and communication difficulties [28].

Dental Care Service Utilization Rate

In Dubai, 65% of children with ASD had visited the dentist, compared to 79.2% of children in the general community, according to research by Mansoor et al. More than one-third (35%) of autistic children had never visited a dentist previously, and 5.3% of parents cited this as their main justification, saying that their child was challenging for the dentist to work with [15].

Discussion

For the benefit of enhancing healthcare and dental treatment for children with ASD, this comprehensive research has drawn a clear picture and highlighted a variety of traits that both serve as barriers to and facilitators of improvement. Several oral health problems and geographic limitations exist for people from challenged groups. This research pinpoints organizational, individual, and policy-level dental barriers, informing decision-makers, stakeholders, and dental specialists about the dental needs of children with ASD.

This evaluation also uncovers several problems with the research, such as a dearth of pertinent studies, poor study designs, a small sample size, inefficient treatments, and a lack of follow-up. There were some intervention studies with sound methodology and clear explanations of the main enablers and barriers, but there were also a few studies with subpar methodology. Numerous studies also fell short of identifying any strategies or methods that were developed for and ineffective against actual standards of oral health.

Low income in families significantly decreased the likelihood that parental caregivers of children with ASD had access to dental treatment in previous years [29]. Furthermore, it might be difficult for parents of children with ASD to afford the necessary dental care if they are struggling economically. Similar conclusions were reached by Wiener et al. [30], who discovered that parents of ASD kids who had financial difficulties did not provide their kids with essential preventative dental care.

Children with ASD pose difficulties for dental professionals, particularly when it comes to acting uncooperatively during oral hygiene procedures [30]. Caregivers of children with ASD said that the children's refusal to participate prevented dentists from treating them [17]. Similar findings were made by Brickhouse et al., who discovered that kids with oral healthcare resistance were assumed to have never seen a regular dentist [31]. Dental practitioners may not want to treat children with ASD because of the difficult behavior they exhibit. Only 32% of general dentists and 89% of pedodontists in the U.S., according to Weil and Inglehart (2010), accept patients with ASD [1]. More proficiency is required for dental personnel on how to handle challenging behavior from clients.

Dental professionals should be more knowledgeable about non-drug options for treating this population because it has been demonstrated that more than half of those diagnosed with ASD need medications taken orally or as a general anesthetic to boost their compliance with dental care [32]. According to caregivers, more than 50% of children with ASD did not receive oral preventive measures to reduce dental phobia [33]. Dental practitioners should receive training in ways to intervene that make it easier for children with ASD to receive dental and oral hygiene care. When using assistive technology, children with ASD are better able to speak, feel less sensitive and frightened, and may be less anxious when getting their teeth cleaned.

The lack of experience dental practitioners have in treating and caring for patients with ASD may prevent them from offering essential oral treatment [19]. Patients with ASD frequently exhibit behaviors such as hyperactivity, anxiety, and hostility, which make it challenging for them to obtain dental care. Elevated sensitivity to sensory input is usually viewed as a care obstacle in the dental environment [30]. Disruptive behavior in patients with ASD can be exacerbated by, among other things, bright lights, uncomfortable objects, loud noises, unfamiliar flavors, and skin contact [34]. Therefore, dental professionals should have specific expertise and a better understanding of the behavioral characteristics of this population to facilitate the treatment of children with ASD.

The excessively long wait for the planned operation was one of the complaints caretakers raised. Children with ASD who are particularly difficult during the perioperative period may act out in several different ways while awaiting admission, during the admissions process, and before surgery [23]. Innovative check-in methods, calm waiting areas, play therapists, and putting ASD children first on the operating room list are all tactics that can reduce problematic behavior.

The cost of treatment and dental health care, the lack of resources, restrictive administrative and system-level policies, the lack of parental demand, community opposition to fluoridation, racial and ethnic differences, inadequate skills, implementation issues, the location of the dental clinic [29], institutionalization, and co-occurring defects were additional significant challenges faced by caregivers of children with ASD. The lack of interaction between dental care and other social or community services, in addition to these issues, represents another ongoing challenge. Our data show that there is a severe lack of dentistry and community service integration on a global scale.

The limitation of the study was that this review was able to include only English-language articles due to a lack of competency in other foreign languages. Grey literature from the year 2000 era, book chapters, case studies, systematic reviews, theses, and articles were also eliminated. Its exclusivity to children with ASD further constrained it.

## Conclusions

If the disparity between unmet dental needs and the use of dental therapy persists, many dental practitioners will find it difficult to address the paucity of solid research regarding the difficulties faced by kids with ASD. Even though there are many obstacles, it seems that the dentist's unavailability and the patient's reluctance to cooperate are the two challenges that come up most frequently. The studies' standards will be enhanced in the future by additional research on the factors that prevent children with ASD from using dental care services, using a standardized questionnaire approach. Also, new technologies and strategies must be tested to establish an effective oral health system. Dental providers could use intervention methods to increase social skills and cooperation among children with ASD in dental clinics.

## References

[1] Barry S, O'Sullivan EA, Toumba KJ (2014). Barriers to dental care for children with autism spectrum disorder. Eur Arch Paediatr Dent.

[2] AlJasser R, Alsinaidi A, Bawazir N, AlSaleh L, AlOmair A, AlMthen H (2023). Association of oral health awareness and practice of proper oral hygiene measures among Saudi population: a systematic review. BMC Oral Health.

[3] Newschaffer CJ, Croen LA, Daniels J (2007). The epidemiology of autism spectrum disorders. Annu Rev Public Health.

[4] Manski RJ, Moeller JF (2002). Use of dental services: an analysis of visits, procedures and providers, 1996. J Am Dent Assoc.

[5] (2024). Data and statistics on autism spectrum disorder. https://www.cdc.gov/autism/data-research/index.html.

[6] Hemdi A, Daley D (2017). The needs of mothers of children with autism spectrum disorder (ASD) in the
kingdom of saudi arabia (KSA): A qualitative study. IJASR.

[7] (2024). U.S. department of health and human services. Healthy people 2020: Oral health. http://www.healthypeople.gov/2020/topics-objectives/topic/oral-health.

[8] India State-Level Disease Burden Initiative Collaborators (2017). Nations within a nation: variations in epidemiological transition across the states of India, 1990-2016 in the Global Burden of Disease Study. Lancet.

[9] Kassebaum NJ, Smith AG, Bernabé E (2017). Global, regional, and national prevalence, incidence, and disability-adjusted life years for oral conditions for 195 countries, 1990-2015: A systematic analysis for the global burden of diseases, injuries, and risk factors. J Dent Res.

[10] Krishnan L, Aarthy CS, Kumar PD (2020). Barriers to access dental care services among the adult population: a systematic review. J Global Oral Health.

[11] Chatterjee P (2017). The health system in India: the underserved majority. Lancet.

[12] Maserejian NN, Trachtenberg F, Link C, Tavares M (2008). Underutilization of dental care when it is freely available: a prospective study of the New England Children's Amalgam Trial. J Public Health Dent.

[13] Haddaway NR, Page MJ, Pritchard CC, McGuinness LA (2022). PRISMA2020: An R package and Shiny app for producing PRISMA 2020-compliant flow diagrams, with interactivity for optimised digital transparency and open synthesis. Campbell Syst Rev.

[14] Alkhthami S (2019). Current Barriers to Dental Care of Virginia Children With Autism Spectrum Disorder (ASD).

[15] Mansoor D, Al Halabi M, Khamis AH, Kowash M (2018). Oral health challenges facing Dubai children with autism spectrum disorder at home and in accessing oral health care. Eur J Paediatr Dent.

[16] Du RY, Yiu CK, King NM, Wong VC, McGrath CP (2019). Oral health behaviours of preschool children with autism spectrum disorders and their barriers to dental care. J Autism Dev Disord.

[17] Taghizadeh N, Heard G, Davidson A, Williams K, Story D (2019). The experiences of children with autism spectrum disorder, their caregivers and health care providers during day procedure: a mixed methods study. Paediatr Anaesth.

[18] Alshihri AA, Al-Askar MH, Aldossary MS (2021). Barriers to professional dental care among children with autism spectrum disorder. J Autism Dev Disord.

[19] Stein LI, Polido JC, Najera SO, Cermak SA (2012). Oral care experiences and challenges in children with autism spectrum disorders. Pediatr Dent.

[20] Nithya T, Shetty P, Sowmya B, Kodgi V (2016). Barriers to dental care for children with autism spectrum disorder-a pilot study. IOSR J Dent Med Sci.

[21] Shapira J, Mann J, Tamari I, Mester R, Knobler H, Yoeli Y, Newbrun E (1989). Oral health status and dental needs of an autistic population of children and young adults. Spec Care Dentist.

[22] Cermak SA, Stein Duker LI, Williams ME, Dawson ME, Lane CJ, Polido JC (2015). Sensory adapted dental environments to enhance oral care for children with autism spectrum disorders: a randomized controlled pilot study. J Autism Dev Disord.

[23] Luppanapornlarp S, Leelataweewud P, Putongkam P, Ketanont S (2010). Periodontal status and orthodontic treatment need of autistic children. World J Orthod.

[24] Jaber MA (2011). Dental caries experience, oral health status and treatment needs of dental patients with autism. J Appl Oral Sci.

[25] Rai K, Hegde AM, Jose N (2012). Salivary antioxidants and oral health in children with autism. Arch Oral Biol.

[26] DeMattei R, Cuvo A, Maurizio S (2007). Oral assessment of children with an autism spectrum disorder. J Dent Hyg.

[27] Chandrashekhar S, Bommangoudar JS (2018). Management of autistic patients in dental office: a clinical update. Int J Clin Pediatr Dent.

[28] Fontaine-Sylvestre C, Roy A, Rizkallah J, Dabbagh B, Santos BFD (2017). Prevalence of malocclusion in Canadian children with autism spectrum disorder. Am J Orthod Dentofacial Orthop.

[29] Alshatrat SM, Al-Bakri IA, Al-Omari WM (2020). Dental service utilization and barriers to dental care for individuals with autism spectrum disorder in Jordan: a case-control study. Int J Dent.

[30] Wiener RC, Vohra R, Sambamoorthi U, Madhavan SS (2016). Caregiver burdens and preventive dental care for children with autism spectrum disorder, developmental disability and/or mental health conditions: National Survey of CSHCN, 2009-2010. Matern Child Health J.

[31] Brickhouse TH, Farrington FH, Best AM, Ellsworth CW (2009). Barriers to dental care for children in Virginia with autism spectrum disorders. J Dent Child (Chic).

[32] Tang SJ, Wei HL, Li CY, Huang MN (2023). Management strategies of dental anxiety and uncooperative behaviors in children with autism spectrum disorder. BMC Pediatr.

[33] Elmore JL, Bruhn AM, Bobzien JL (2016). Interventions for the reduction of dental anxiety and corresponding behavioral deficits in children with autism spectrum disorder. J Dent Hyg.

[34] Junnarkar VS, Tong HJ, Hanna KM, Aishworiya R, Duggal M (2023). Qualitative study on barriers and coping strategies for dental care in autistic children: parents' perspective. Int J Paediatr Dent.

